# Genome-wide mapping of DNase I hypersensitive sites and association analysis with gene expression in MSB1 cells

**DOI:** 10.3389/fgene.2014.00308

**Published:** 2014-10-13

**Authors:** Yanghua He, Jose A. Carrillo, Juan Luo, Yi Ding, Fei Tian, Irit Davidson, Jiuzhou Song

**Affiliations:** ^1^Department of Animal and Avian Sciences, University of MarylandCollege Park, MD, USA; ^2^Division of Avian Diseases, Kimron Veterinary InstituteBet Dagan, Israel

**Keywords:** DNase I, DHS, intergenic DHSs, MSB1, CpG islands, gene expressions, long non-coding RNAs, Marek's disease (MD)

## Abstract

DNase I hypersensitive sites (DHSs) mark diverse classes of cis-regulatory regions, such as promoters and enhancers. MSB-1 derived from chicken Marek's disease (MD) lymphomas is an MDV-transformed CD4+ T-cell line for MD study. Previously, DNase I HS sites were studied mainly in human cell types for mammalian. To capture the regulatory elements specific to MSB1 cells and explore the molecular mechanisms of T-cell transformation caused by MDV in MD, we generated high-quality of DHSs map and gene expression profile for functional analysis in MSB1 cell line. The total of 21,724 significant peaks of DHSs was identified from around 40 million short reads. DHSs distribution varied between chromosomes and they preferred to enrich in the gene-rich chromosomes. More interesting, DHSs enrichments appeared to be scarce on regions abundant in CpG islands. Besides, we integrated DHSs into the gene expression data and found that DHSs tended to enrich on high expressed genes throughout whole gene regions while DHSs did not show significant changes for low and silent expressed genes. Furthermore, the correlation of DHSs with lincRNAs expression was also calculated and it implied that enhancer-associated lincRNAs probably originated from enhancer-like regions of DHSs. Together, our results indicated that DNase I HS sites highly correlate with active genes expression in MSB1 cells, suggesting DHSs can be considered as markers to identify the *cis*-regulatory elements associated with chicken Marek's disease.

## Introduction

The formation of regions of open chromatin or nucleosome loss in eukaryotic genomes is a vital factor revealing potential regulatory activity. In addition, chromatin accessibility, which has been determined traditionally by regions of “open” or “closed” conformation, is governed by accessible *cis*-regulatory elements from DNA sequence, ATP-dependent chromatin remodeling and nucleosome modifications (Bell et al., 2011). However, chromatin accessibility can be examined by DNase I cleavage digestion, and then disclosed by the DNase I cleavage pattern (Wu et al., 1979). The introduction of next generation sequencing technology triggered one of the major breakthroughs in genomic research. The combination of DNase I digestion and deep sequencing (DNase-seq) has been used to reveal chromatin accessibility *in vivo* in a specific tissue or cell-type on a genome-wide scale (Song and Crawford, 2010).

Identification of the causative agent of Marek's disease (MD) had long been the holy grail of MD research and the highly contagious Marek's disease virus type 1 (MDV-1) is an avian herpesvirus that causes T-cell lymphomas and mononuclear infiltration of peripheral nerves (Luo et al., 2012). However, the molecular mechanisms that underlie T-cell transformation caused by MDV are unknown. MSB-1 is an MDV-transformed CD4+ T-cell line derived from a spleen lymphoma induced by the BC-1 strain of MDV-1 (Akiyama and Kato, 1974; Hirai et al., 1990). Therefore, the MSB-1 lymphoblastoid cell line, which shares many properties of Marek's disease (MD) tumors, could be used as a model system for analyzing the molecular pathways and mechanisms of neoplastic transformation in MD tumors. It was known DNase I HS sites are specific for different cell types and tissues (Crawford et al., 2006). In the previous studies, the exploration of chromatin accessibility and recognition of gene regulatory elements by DNase-seq technique were conducted mostly in human or mouse cell types for mammalian. However, genome-wide analysis of DNase I hypersensitive sites in chicken has not been reported yet. Hence, our study is to explore the regulatory pattern of DNase I hypersensitive sites in chicken MSB1 cell line, so as to probe molecular mechanisms of T-cell transformation caused by MDV in MD development.

In the present research, we enriched cleavage fragments of DNA treated with DNase I (200–500 bp) and constructed a DNA sequencing library from chicken MSB1 cell line. From 45,960,000 DHS sequencing reads, 21,724 DHSs were identified with high sensitivity. By combining the genome-wide analysis of DHS and gene expression sequencing, we found a specific correlation between DHS locations and gene expressions in MSB1 cells. Our data suggested DNase I hypersensitive sites provide vital clue to identify *cis*-elements for active genes expressions.

## Methods

### Preparation of DNase I treated DNA

The MDV-transformed lymphoblastoid MSB-1 cells were obtained from Dr. Mary Delany's lab, University of California, Davis, CA. and grown at 38.5°C in 5% CO_2_ in RPMI 1640 medium containing 10% fetal calf serum, 10% tryptose phosphate broth, and 1% sodium pyruvate (Yao et al., 2008). Intact nuclei were prepared and digested with DNase I (He et al., 2012). Briefly, cells were lysed with 0.1% NP40 and nuclei were collected by centrifugation. Intact nuclei were treated with DNase I amounts of 0 units (U), 1 U, 5 U, 40 U, and 80 U per 200 μl reaction at 37°C for 5 min, and reactions were stopped with 0.1 M EDTA. Optimal concentrations of DNase I generated a smear of high-molecular-weight fragments when analyzed by pulsed field gel electrophoresis. The fragments of 200–500 bp were cut from the gel and DNA was extracted using the standard phenol-chloroform technique.

### DNA library preparation and high-throughput sequencing

The library for sequencing on the Solexa 1G Genome Analyzer (Illumina, USA) was constructed as follows. End repair of the fragmented DNase I treated DNA was performed by NEBNext® End Repair Module (NEB, MA, USA). Then a 3′ polyA was added using DNA polymerase I, Large (Klenow) Fragment (NEB, MA, USA). Also, a pair of Solexa adaptors (Illumina, USA) was ligated to the repaired ends by T4 ligase (Promega, USA). Filtration in a 2% agarose gel was used to select fragments (DNA plus adaptors) from 200 to 500 bp. PCR was conducted to enrich purified DNA fragments by using Phusion® Hot Start High-Fidelity DNA Polymerase (NEB, MA, USA). After purification, DNA quality was examined by using the Qubit assay (Life Technology, USA) and was diluted for sequencing, then we performed sequencing analyses in the Solexa 1G Genome Analyzer (Illumina, USA) following manufacturer protocols.

### Alignment and peak identification of DNase I HS sites

Sequence reads of 50 bp length of DNase-seq were obtained using the Solexa Analysis Pipeline. And then they were mapped to the chicken reference genome by Bowtie and only perfect matches that had a single unique alignment within the genome were retained and used for further analysis. For DNase-seq experiment, peak areas represent *in vivo* locations of DNase I hypersensitive sites. The WaveSeqR package that employs robust method based on the wavelet transformation was applied to identify DNase I peaks (Mitra and Song, 2012). The parameters configuration was window size of 200 bp, minreads of 3, maxscale of 12, the wavelet mother function of “gaussian2,” no gap and p.thres of 0.2 to call peaks representing putative DNase I hypersensitive sites. The output result includes the genome coordinates, reads number of each peak, *p*-value, and FDR. Furthermore, for peak related genes, as long as there is 1 bp overlap between regions of a peak and a particular gene (includes regions of up-2 K, exon, intron and down-2 K), we consider that the peak is associated with the correspondent gene. The pathway analysis of genes relevant peaks was conducted by DAVID database. To link the DNA methylation and DNase I HS sites, CpG islands information about chicken was downloaded from the UCSC website (http://hgdownload.cse.ucsc.edu/goldenPath/galGal3/database).

### Whole genome gene expression analysis

The total RNA extraction was performed by RNeasy Mini Kit (Qiagen, Valencia, CA, USA) from prepared MSB1 cells. Isolation of mRNA from total RNA was achieved using Oligotex mRNA Mini Kit (Qiagen, Valencia, CA, USA) according to the manufacturer's instruction. About 300 ng of mRNA was used to synthesize the first strand cDNA by SuperScriptTM II Reverse Transcriptase (Invitrogen, Frederick, MD, USA). The second strand cDNA was synthesized using DNA polymerase I (Invitrogen, Frederick, MD, USA) with addition of Ribonuclease H (Invitrogen, Frederick, MD, USA) to degrade the remaining mRNA. After purification, a Bioruptor Sonicator (Diagenode, NJ, USA) fragmented the double strand cDNA (dscDNA) to approximately 200–500 bp. Then the library was built for sequencing on the Solexa 1G Genome Analyzer (Illumina, USA) following manufacturer protocols.

The total number of tags that uniquely aligned to gene represented its expression level. And the unique mapped tags for each gene were normalized to TPM (number of transcript copies in per million clean tags), equaling to the copy number of clean tags for this gene divided by total number of clean tags and multiplied by one million for multiple samples comparison (Morrissy et al., 2009). Normalized gene expression levels were averaged with two biological duplicates for each gene.

### Correlation of DHSs to gene expression

To study the correlation of DNase I hypersensitive sites with gene expressions, transcriptional levels of genes in chicken MSB1 cells were obtained by RNA-seq analysis. Then, these genes (17,934 genes) were broken up into 170 sets of 100 genes by ranking their expression levels. Four out of the 170 sets shown in **Figure 4** correspond to highly expressed, two degrees of intermediately expressed (medium and low) and silent genes respectively. Tags detected were aligned in each gene set across transcription start sites (TSS) or gene bodies. To calculate the DHSs profiles across the gene bodies, the tag numbers detected in every 5% of the gene-body region and every 1 kb outside of the gene-body region were summed and normalized in the four expressed sets. For DHSs analysis near TSS (**Figure 5**), the tag density (number of tags per base pair) was calculated in the top 1000 high expressed and 1000 low expressed genes relative to the upstream 100 Kb of TSS.

### Validation of DHSs by real-time PCR

DNase-qPCR reactions with SYBR green dye were carried out using BIO-RAD MyiQ qPCR machine to confirm the enrichment of selective putative DHSs regions. PCR primer pairs were designed using Primer3 (http://fokker.wi.mit.edu/primer3/input.htm) and confirmed by Oligo 6. Primer sequences were given in Table S3. The DNase-qPCR reactions were triplicated for each site. To determine the relative fold enrichments, the 2^−ΔCp^ method was used by comparing enrichment values for a positive primer pair (totally 5 pairs) to a negative primer pair between experimental (DNase DNA) and reference (input DNA) samples. For RT-qPCR of gene expression, five candidate genes were selected to validate the association with DHSs and triplicates were performed for RT-qPCR reactions. Gene expression was normalized against *GAPDH* housekeeping gene in the corresponding samples.

## Results

### Distribution of DHSs reads

To identify regions of the genome where regulatory factors interact with DNA to modify chromatin structure and gene transcription, DNase-seq has been employed to map regulatory regions in MSB1 cell line. A total of 55.93 and 35.99 million short reads from two biological duplicates were aligned to the chicken reference genome with unique mapping rates of 80.50 and 80.29%, respectively.

**Figure 1:**
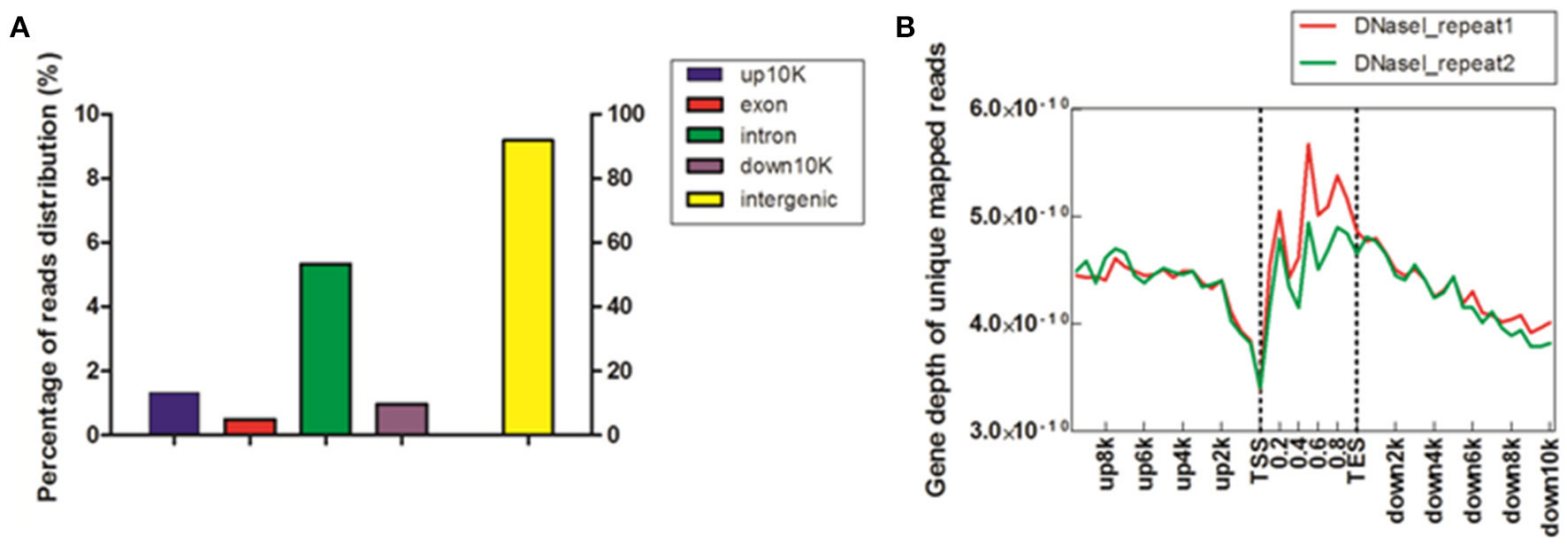
**Distribution of DHSs reads in MSB1 cells**. **(A)** Distribution of unique mapped reads among different genomic regions. The chicken genome was divided into five kinds of regions: 10 kb upstream of transcription start site (TSS), exon, intron, 10 kb downstream of transcription end site (TES) and intergenic regions. The histogram described the percentage of unique mapped reads among five genomic regions. **(B)** Coverage depth of unique mapped reads among genic regions. For each gene, the tag numbers detected in every 10% of the gene-body region and every 5000 bp outside of the gene-body region were summed to obtain density levels. These numbers were then normalized by the total number of base pairs in each region (Barski et al., 2007).

To study DHSs distribution regarding genomic region, we divided the chicken genome into five kinds of regions –up-10 K [10 kb upstream of transcription start site (TSS)], exon, intron, down-10 K [10 kb downstream of transcription end site (TES)] and intergenic regions–based on the annotation of “known genes” from UCSC galGa3 database. The reads proportion for each region of the entire genome was indicated (Figure 1A). As shown in Figure 1A, the majority of reads were assigned to intergenic regions (91.8%) and only a few reads to exonic sections (0.49%). Intronic region constituted 5.34% of the mapped reads and it was approximately ten times higher than for exon region. Further, percentage of reads was 2–3 times higher in upstream and downstream regulatory regions than in exon region. To visualize the distribution trends of DHSs in the gene regions, a composite profile of DHSs for all known genes was generated, spanning their gene bodies and extending it 10 kb upstream and 10 kb downstream (Figure 1B). It is notable that the levels of DHS signals were high on gene body regions. Moreover, it appeared that DHSs decreased dramatically at TSS, suggesting that DHSs specifically concentrate in regions proximal to TSS. These results were consistent with previous observations that unique mapped reads of DNase I increased around TSS in HeLa S3 cells (Wang et al., 2012). Besides, our results in Figure 1B also showed that more DHSs were enriched in upstream regions of TSS comparing to downstream regions of TES, implying that DHSs could explore some *cis*-regulatory elements, such as enhancers acting on the promoter regions via bounding by activator proteins (Pennacchio et al., 2013).

### Distribution of DHSs peaks

**Figure 2:**
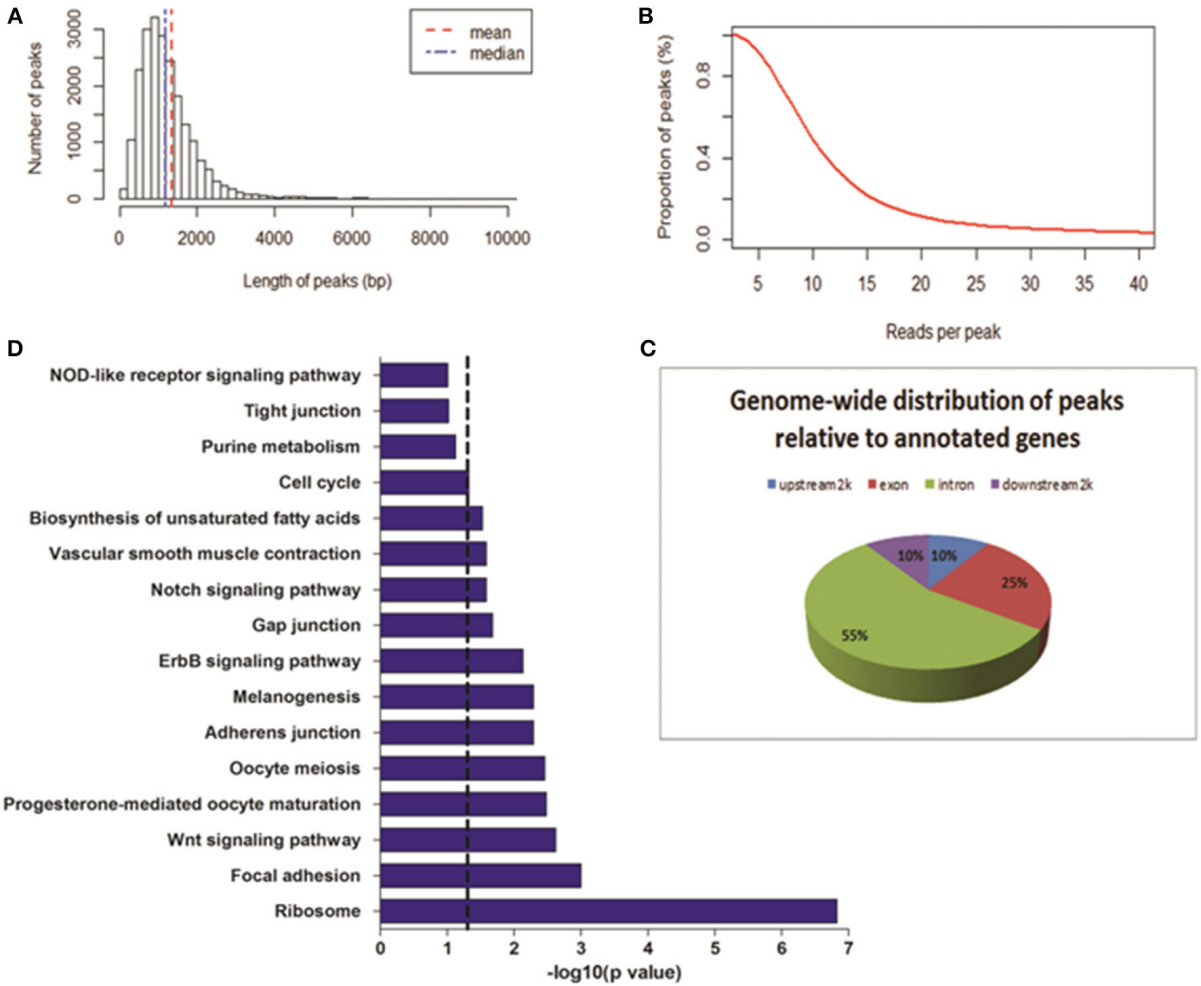
**Genome-wide distribution of DNase-seq peaks**. **(A)** Length of peaks. X-axis represents the length of peak; Y-axis represents the number of peaks. **(B)** Proportion of peaks with reads number. Coverage of reads in peak regions was calculated. The reads number of each peak and peak numbers were added with the cumulative density statistics. **(C)** The locations of DHSs relative to annotated genes. Genome-wide distributions of DHS peaks in annotated gene regions were shown. DHS peaks were counted in upstream 2 Kb, exon, intron, and downstream 2 Kb regions. **(D)** Pathways analysis of genes related to DHS peaks. Dashed line: threshold line corresponds to *P*-value of 0.05.

To determine the DNase I hypersensitive sites within the genome of MSB1 cells, a more robust method, WaveSeqR software, was adopted to accurately identify enriched regions of DNase I HS sites (Mitra and Song, 2012). Statistically, the total of 21,724 DHSs peaks was identified (*p*-value <0.2). The average and median peak length were 1335 and 1199 bp correspondingly (Figure 2A). The reads numbers of peaks and the peaks counts were calculated using cumulative density statistics. Most of peaks can be identified by around 20 reads in MSB1 cells (Figure 2B). To study the pattern of DNase I hypersensitive sites in different regions of genes, we also calculated the distribution of peaks in four kinds of genic regions, most peaks (55%) were enriched in intronic region, 10% of them were accounted in upstream-2 kb and downstream-2 kb regions, respectively (Figure 2C). The results differ from the patterns of DHSs in HeLa cells, in which more reads were found in upstream 20 kb and downstream 20 kb than in coding region (Wang et al., 2012). In addition, we also found 4465 genes were associated with DHSs peaks, and they actively involved into many biological processes, such as protein amino acid phosphorylation and intracellular signaling cascade, the molecular functions of nucleotide binding and ribonucleotide binding (Table S1). Further pathway analysis demonstrated most of genes related to DHSs involved into ribosome, focal adhesion and Wnt signaling pathway (Figure 2D).

### Distribution of DHSs on different chromosomes

**Figure 3:**
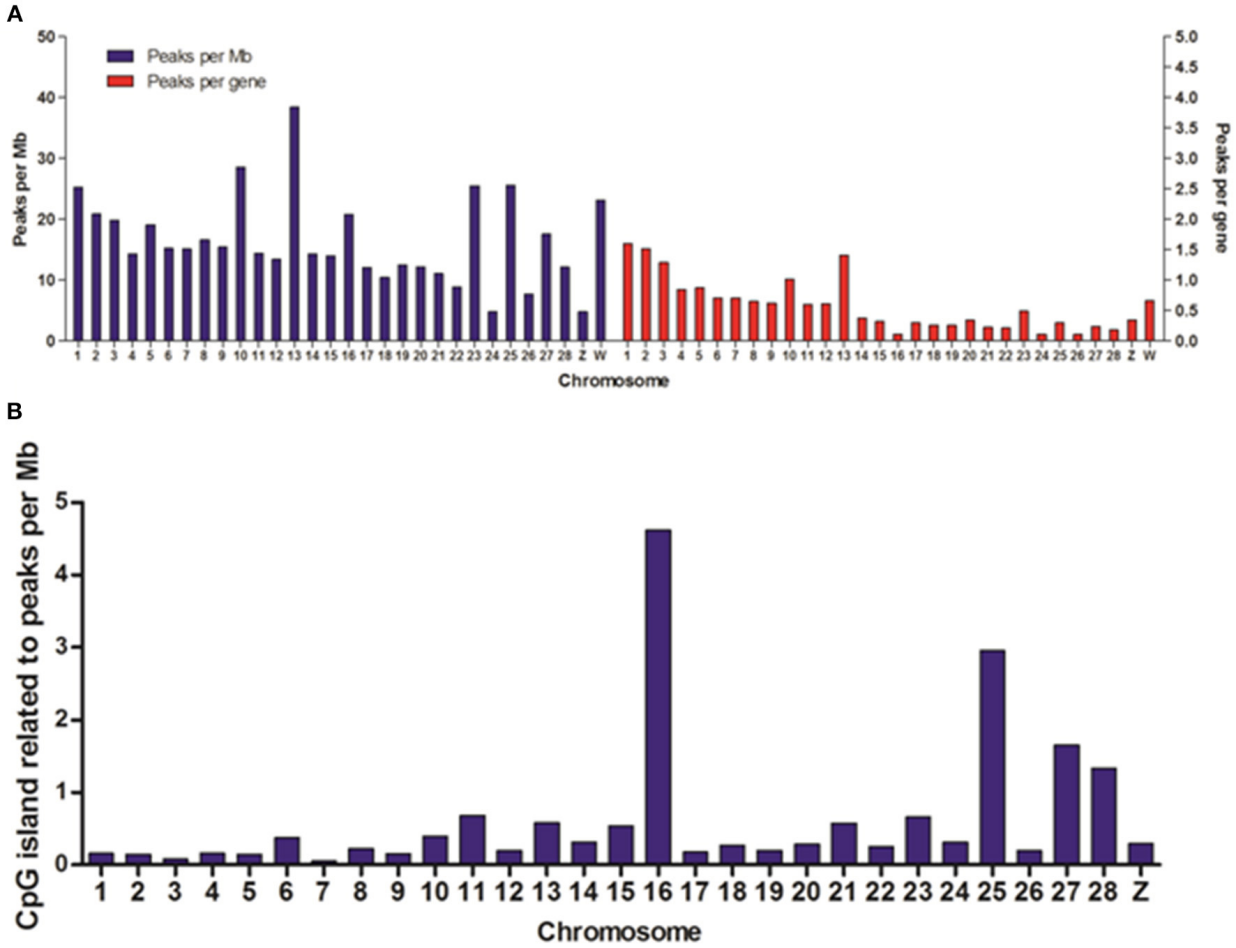
**Distribution of DHSs and CpG islands-associated DHSs on different chromosomes**. **(A)** Distribution of DHSs on the annotated genome. DHS peaks were mapped to each chromosome. The peak densities for per Mb (blue bars) and for per gene (red bars) were calculated. **(B)** The densities of CpG island-related peaks for per Mb on different chromosomes. We ignored the characteristics of DHSs on all of random chromosomes.

To reveal the difference of DHSs distribution among chromosomes, we mapped the locations of DHSs relative to chromosomes, annotated genes and CpG islands. We found that DHSs peaks were significantly over-enriched on chromosomes 1, 2, 3, 10, 13, 23, 25, and W, which are known to be especially gene-rich (Figure 3A). Besides, the density of DHSs peaks per gene varied among chromosomes and they were highly enriched on these chromosomes. Notable, there were more DHS peaks on chromosome 16 while peaks density per gene was very low, this may be due to smaller chromosome size.

DNA methylation is one of the most prevalent mechanisms to maintain inactive genomic regions in a repressed state, and it is also one of the most stable modifications (Bird, 2002). In order to study the relationship between DNA methylation and DNase I hypersensitive sites, we overlapped DHSs peaks with CpG islands and normalized per Mb in chromosomes except for all random chromosomes. The results showed chromosomes 1, 2, 3, 10, 13, 23, and W highly enriched DHSs peaks per gene appeared to low density of CpG islands (Figure 3B), conversely, there was significant high density CpG islands on chromosome 16 when DHSs peaks density per gene was very low on this chromosome, which suggested that DNase I sensitive domain preferred to act within active chromatin domains that present low density CpG islands (Cockerill, 2011).

### Overall correlation between DHS distribution and gene expression

To reveal the functional consequences of DNase I hypersensitive sites, gene expression profiles were generated by the next-generation sequencing in MSB1 cells. The number of unique mapped reads for each gene was counted and then normalized to TPM (number of transcript copies in per million clean tags) to represent gene expression levels (Morrissy et al., 2009).

**Figure 4:**
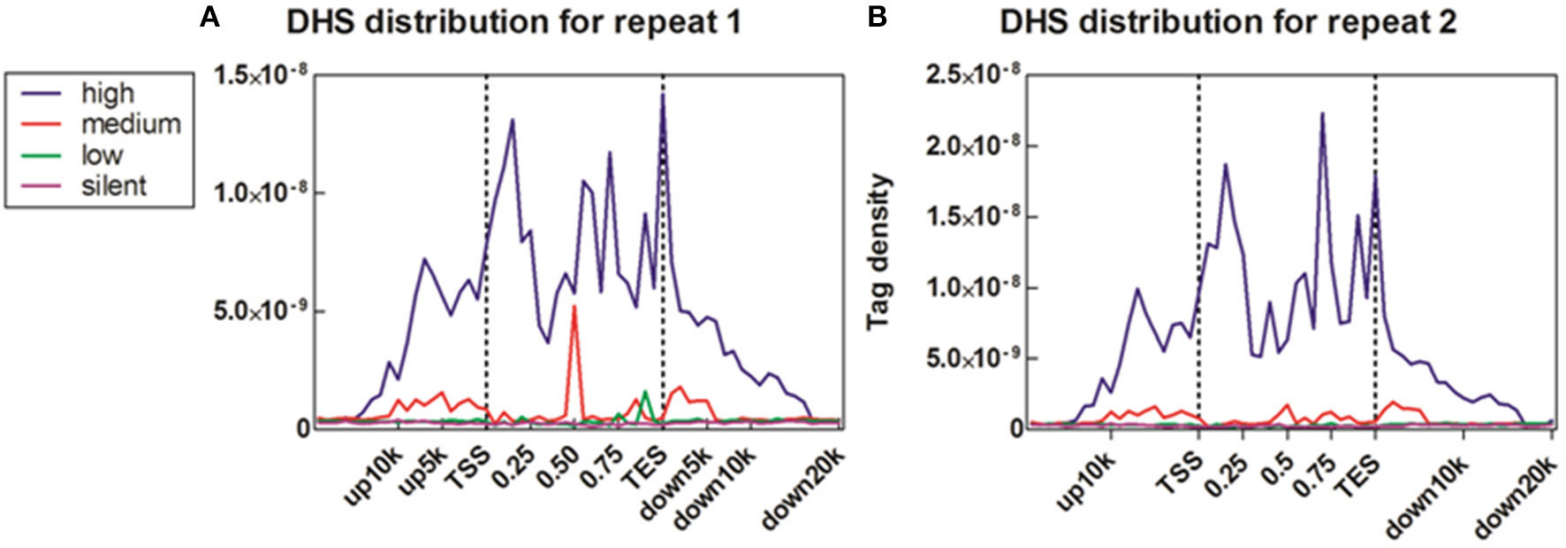
**Correlation between DHS distribution and gene expression**. **(A)** Profiles of DHSs distribution patterns were shown across the gene bodies for highly active (high), two kinds of intermediately active (medium and low) and silent gene sets. Each gene set included 100 genes according to their expression levels in MSB1 cells line. Here, DHSs reads were aligned extending 20 kb of 5′ and 3′ of the gene bodies of 100 genes in each group (x axis). The y axis shows the detected tag density. For each gene, the tag numbers detected in every 5% of the gene-body region and every 1 kb outside of the gene-body region were summed to obtain DHSs distribution levels. These numbers were then normalized by the total number of base pairs in each region. **(B)** Profiles of DHSs distribution patterns in sample repeat 2 of MSB1 cells.

**Figure 5:**
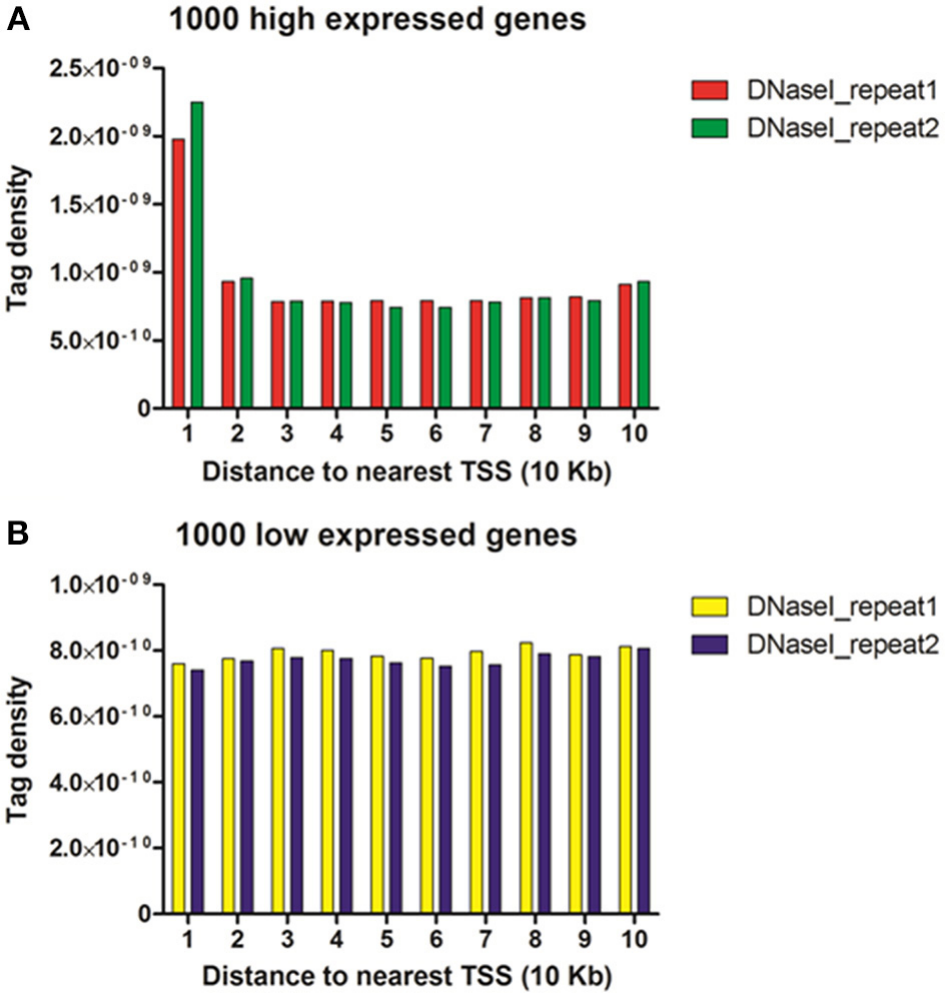
**DHSs reads were enriched near genes that were expressed in the MSB1 cells**. **(A)** DHSs density levels in upstream 100 Kb regions relative to TSS for 1000 high expressed genes. **(B)** DHSs density levels in upstream 100 Kb regions for 1000 low expressed genes.

To reveal the DNase I regulation pattern in MSB1 cells, the genes whose expression levels were determined by the RNA-seq assay were attributed to multiple groups. Four groups were selected randomly with 100 genes for each group according to their expression levels. The DNase I reads numbers in each region were calculated and normalized throughout the whole transcribed regions and extending 20 kb upstream and 20 kb downstream for four gene sets corresponding to highly expressed, two types of intermediately expressed (medium and low) and silent genes (Figure 4). As expected, DNase I hypersensitive sites signals were correlated with gene activation (Figure 4). Obviously, DHSs enrichment levels were superior at high expressed genes than at low expressed and silent genes. Intriguingly, DHSs levels were elevated surrounding the TSSs and TES for the highly expressed genes sets (dotted line), though were not significant for the other three sets. To explore DHSs features in extreme high and low expressed genes and reveal the association of DHSs with *cis*-regulatory elements on upstream regions relative to TSS, we analyzed the density levels of DHSs in extending 100 kb upstream for two sets genes with the top 1000 high expressed and 1000 low expressed genes. The result showed that the most pronounced enrichment was observed within 10 kb upstream of promoters of high expressed genes. DHSs enrichment levels appeared to decrease while increasing the distance from TSSs (Figure 5A). In contrast, DHS sites enrichment did not change within 100 kb upstream of TSSs in low expressed genes (Figure 5B). These observations were consistent with p300 binding sites that a near-ubiquitously expressed component of enhancer-associated protein assemblies drive the expression of adjacent genes in forebrain tissue isolated from mouse embryos at given time point (Visel et al., 2009), suggesting that DNase I hypersensitive sites have a strong relationship with enhancer regulatory element in chicken MSB1 cells.

### DHSs and long non-coding RNA

**Figure 6:**
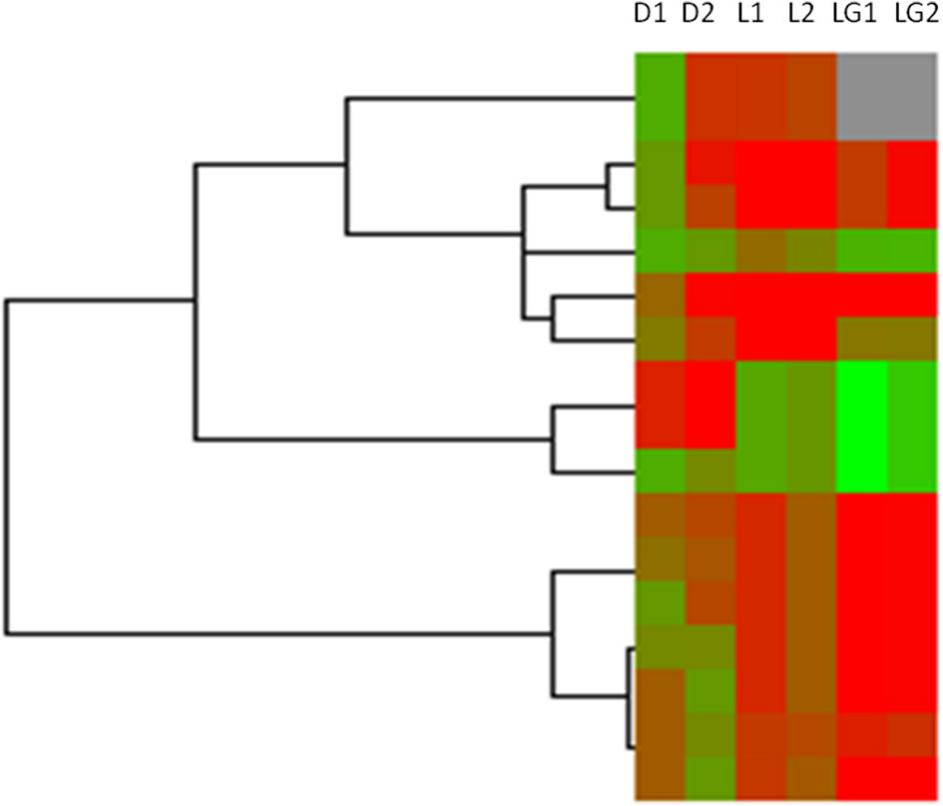
**Heatmap of DHSs, overlapped lincRNAs and neighboring genes of lincRNAs in MSB1 cell line**. Based on RNA-seq data in MSB1 cell line, 124 candidate lincRNAs were identified and only 17 lincRNAs are overlapped with DNase I hypersensitive sites (DHSs). Normalized intensity values of DHSs (rows) and expressions values of their overlapped lincRNAs and neighboring genes of lincRNAs were ordered using Centroid Spearman Rank Correlation and hierarchical clustering in Cluster3.0 software. The intensity of DHS was represented by reads number in this peak of DNase I hypersensitive site. The expression of lincRNA and its neighboring genes were represented by FPKM (Fragments Per Kilobase of transcript per Million mapped reads). The dendrogram showed the similarity (distance) of DHSs intensity, lincRNAs and neighboring genes expressions and was divided into sub-trees as distinguished from different colors. Arrays (columns) were grouped that *D1* stands for the intensity of DHSs in duplicate 1, *D2* stands for the intensity of DHSs in duplicate 2, *L1* stands for expressions of lincRNAs overlapped DHSs in duplicate 1 and *L2* stands for expressions of lincRNAs overlapped DHSs in duplicate 2; *LG1* and *LG2* are expressions of neighboring genes of lincRNAs in duplicate 1 and duplicate 2, respectively. Red and green colors reflect the high and low intensities, respectively.

It has been reported that some long non-coding RNAs (lncRNAs) originate from intragenic enhancers which behave as alternative promoters producing transcripts when active (Marques et al., 2013). Accordingly, our results showed that abundant DHSs were enriched in intergenic region in MSB1 cells (Figure 1A). Therefore, in order to determine whether lncRNAs might originate from active intergenic enhancers examined by DHSs, we also analyzed the distribution of DHSs relative to long intergenic non-coding RNAs (lincRNAs) in MSB1 cell line, based on a stringent lincRNA identification pipeline for RNA-seq data from our lab (unpublished). We found 124 candidate lincRNAs, nonetheless, only 17 of those overlapped with intergenic DHSs (Table S2). These observations indicated that DHSs may possibly be less important as regulatory elements for non-coding RNA genes than for coding genes, which was consistent with information from *C. elegans* (Shi et al., 2009). However, an enhancer examined by DHS sites might affect gene transcription not only in *cis* and it can be found within introns or even be excised and inserted elsewhere in the chromosome and still affect gene transcription (Eichenlaub and Ettwiller, 2011). Therefore, we explored whether DHSs as enhancer regulatory elements regulate lincRNAs expressions by calculating the correlation between the enrichments of DHSs and the expressions of overlapping lincRNAs (Figure 6). It showed a negative correlation between enrichment of DHS and lincRNA expression. To test whether lincRNAs were co-expressed with protein-coding neighbors, Pearson correlations of expression levels between lincRNAs and neighboring protein-coding genes were also calculated (Figure 6). We observed that lincRNAs affect their neighboring protein-coding genes but there is no stable mode, which is similar to previous studies in human and zebrafish (Cabili et al., 2011; Pauli et al., 2012). Therefore, DHSs enhance gene expression and the expression of lincRNAs is associated with the expression of enhancers but act not completely in *cis*.

### Validation of DHSs by real-time PCR

**Figure 7:**
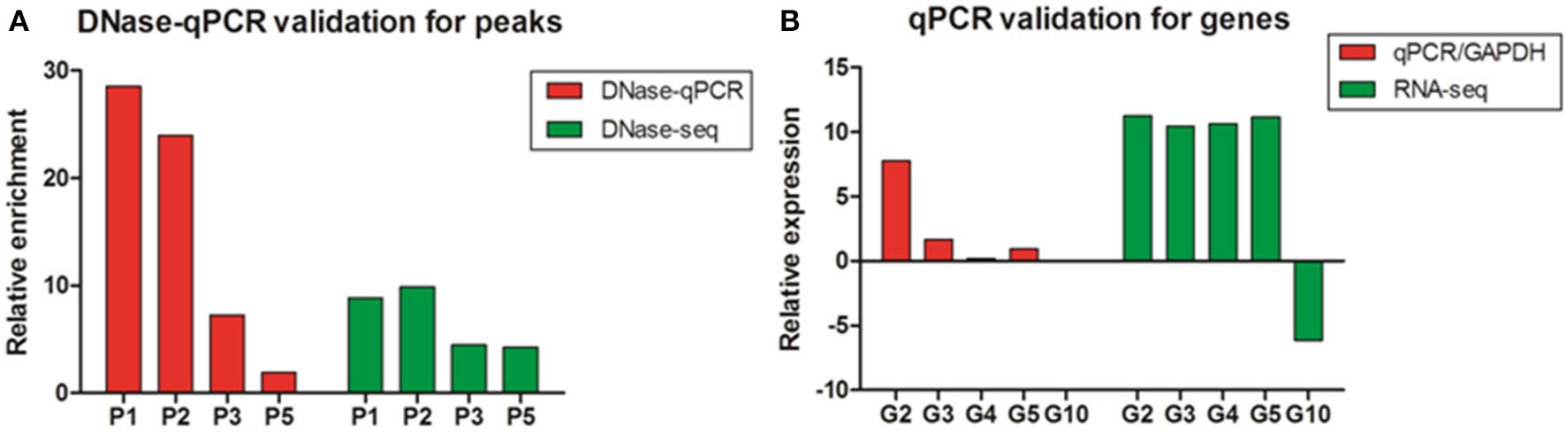
**The validation of DNase I hypersensitive sites and gene expression in chicken MSB1 cells**. **(A)** The validation of DHSs peaks by DNase-qPCR. Real-time PCR results showing enrichment of indicated four sites (P1, P2, P3, and P5) in DNase-seq results were carried out in MSB1 cells (red bar). The negative control was selected from regions without DHSs coverage in the whole genome in both of replicates to normalize the relative enrichment levels of DNase I hypersensitive sites. DNase-seq results were also shown by the logarithm base 2 values of average reads number of DNase I in MSB1 cells (green bar). **(B)** Real-time RT-PCR were performed for validation of genes expression and standardized with *GAPDH* housekeeping gene (red bar). RNA-seq result for each selected gene was also shown and the logarithm base 2 values of TPM were used as expression levels (green bar). For G10 gene, it is difficult to show due to very low expression (0.00265).

To assess the accuracy of the DNase-seq mapping results and confirm the relationship between DNase I HS sites and the expressions of related genes, five DHSs peaks overlapped with neighboring genes, including high expressed and low expressed correspondents, were arbitrarily chosen to confirm their enrichment using DNase I—quantitative PCR (DNase-qPCR) approach. Relative enrichment was quantified for each site with real-time PCR reactions using 0.5 ng DNase I treated DNA or 0.5 ng input DNA and normalized by the negative control without DHSs coverage. For the four candidate peaks of DHSs (Figure 7A), the relative enrichments were mostly consistent with DNase-seq profiles. Similarly, the expression levels of genes related to DHSs peaks were also detected with reverse transcription— quantitative PCR (RT-qPCR) and standardized with the *GAPDH* housekeeping gene. The results showed that the expressions of five genes were predominantly consistent with the RNA-seq data (Figure 7B) and the enrichment value of G10 gene was significant low. Besides, G2, G3, and G5 are genes overlapping with P2, P3, and P5 peaks, successively (Table S3), and it showed expressions of the neighboring genes to DHSs would decrease with the enrichment levels of DHSs sites declined, which implied DHSs are indeed associated with active genes and they probably represent regulatory elements (e.g., enhancers) to drive adjacent genes expressions. Consequently, DNase-seq can be reliably and efficiently used for revealing chromatin accessibility and identifying important regulatory elements.

## Discussion

DNase-Seq (DNase I hypersensitive sites sequencing) is a method used in molecular biology to identify the location of regulatory regions, based on the genome-wide sequencing of regions super sensitive to cleavage by DNase I (Crawford et al., 2006). Finding peaks from DNase-seq is the main goal to identify the location of candidate regulatory regions. However, the lack of well-established algorithms to handle DNase-seq data and the utilization of a ChIP-seq peak finder which does not completely fit the pattern of the DNase-seq data, inspired us to develope a WaveSeqR method that can be used accurately for both narrow and broad peaks (Mitra and Song, 2012). For the implementation of WaveSeqR, we set gaussian2 as the wavelet mother function that is suitable for diffuse peaks of DNase I hypersensitive sites whereas Morlet mother function is good for sharp and punctate peaks (e.g., TFBS and H3K4me3).

Consequently, 21,724 broad significant peaks of DHSs were identified within the genome of MSB1 cells. To compare the accuracy of different methods for identifying DHSs sites in MSB1 cells, the conventional software MACS (version 1.4.2) was also implemented to find candidate DHSs. The total of 30,834 and 16,669 DNase I HS sites were identified from two replicates, respectively. Of those DHSs, 9911 peaks (*p*-value < 1e−05) were common between two replicates of MSB1 cells. Average and median peak length were 205 and 173 bp respectively (Figure S2). After comparing the WaveSeqR and MACS results, 45% of the peaks (4497) were identified by both methods, which suggested that those DHSs are reliable candidates for DNase I hypersensitive sites. Also, WaveSeqR can be considered an accurate and reliable method for the identification of DNase I hypersensitive sites based on DNase-seq data.

From the Figure 1A, we can see that most DHSs reads were allocated to intergenic and intronic regions, however, the percentages of these regions were also greater than others in the whole genome. Therefore, we normalized the DHSs reads distribution to DHSs abundance based on the percentage value of various functional regions (Figure S1). Similarly, reads abundance in intergenic region was still the highest (1.29), followed by exonic region (0.40) and intronic region (0.36), which suggested an orderly preference of DNase I for those genomic sections. Several studies showed that various macrophage-specific DHSs were identified within mouse intron 2. The sequences of those DHSs are highly conserved and some of them can be denoted as intron regulatory elements, such as FIRE, acting as a macrophage-specific enhancer in the *fms* gene expression (Himes et al., 2001). Additionally, it has been reported that 95% of DHSs were observed in intronic and intergenic regions based on 125 different human cell types (Thurman et al., 2012). Therefore, DHSs located in introns and intergenic regions would like to be expected to the vital and conserved regulatory elements without influencing by cell-type and tissue-type specific.

Abundant DHSs were enriched in intergenic region of MSB1. This finding accords with previous studies where approximately half of the DHSs were mapped to intergenic regions in *C. elegans* and were allocated far from annotated genes denoted transcriptional regulatory information (Shi et al., 2009). Moreover, it has been reported that nematode highly conserved non-coding elements (CNEs) were associated with *cis*-regulatory elements (Vavouri et al., 2007). Also it has been reported that DHSs and particularly distal intergenic DHSs, tend to fall in genomic sections that are conserved in two distinct nematode genomes, which implied that conserved DHSs would help to determine what type of functional elements these regions might represent. Our results implied that there was a strong relationship between DHSs enrichments and lincRNAs expressions, and these enhancer-associated lincRNAs probably originated from enhancer-like regions of DHSs (Marques et al., 2013).

The profiles of DNase I hypersensitive sites were determined employing the DNase-seq method on MSB1 chicken cells. Our data showed that most DHSs enriched in intronic, intergenic and upstream regulatory regions. Probably, the intronic and intergenic DHSs are vital and conserved regulatory elements regardless cell or tissue types. By the combination of DNase-seq and RNA-seq analyses in MSB1 cells, the function of DNase I HS sites was explored and showed that they were correlated with active genes, especially high expressed genes, implying that DHSs are potential representatives of enhancer regulatory elements. Even though the information of DNase I HS sites in MSB1 cell line provided an important reference for chicken Marek's disease study, it is still necessary to conduct DNase-seq in different cells or tissues, or different states of the same tissue, including normal vs. Marek's disease infected, to identify global changes in regulation. The method of DNase-seq can help to recognize the functional regions of the genome, however, determining the type of regulatory function for each DNase I hypersensitive site still remains a daunting challenge. Clues can be gleaned from correlating DNase I hypersensitive sites with sequence conservation, promoter or enhancer activity, transcription factor binding sites and histone modifications, motif discovery, DNA methylation and more detailed gene expression analysis. Therefore, in the near future, the integrated analysis of genes, regulatory elements and chromatin architecture on a genome-wide scale will be a powerful and well-established method for identifying functional and regulatory elements.

## Author contributions

Yanghua He: designed the experiments, analyzed the data and wrote and revised the manuscript; Jose A. Carrillo: analyzed the data; Juan Luo and Fei Tian: implemented the DNase-seq experiment and constructed the sequencing libraries; Yi Ding: validated the work of this study; Irit Davidson and Jiuzhou Song: designed the project, interpreted the data and the results, revised the manuscript.

### Conflict of interest statement

The authors declare that the research was conducted in the absence of any commercial or financial relationships that could be construed as a potential conflict of interest.
