# Supplementary material for: Genome-wide mapping of DNase I hypersensitive sites and association analysis with gene expression in MSB1 cells
Source: Front Genet. 2014 Oct 13;5:308. doi: 10.3389/fgene.2014.00308 (PMC4195362; doi:10.3389/fgene.2014.00308)
Supplement: Supplementary file 1 [file DataSheet1.DOCX]

**Table S1. GO enrichment analysis of DHSs peak-related genes.** In each of ontology that biological process, cellular component and molecular function, top 10 significant GO terms were shown. The number and the proportion of GO-related genes and p value were included for each term by DAVID Bioinformatics Resources 6.7, NIAID/NIH.

| \| Category \| Term \| Number of genes \| \| Percent of genes (%) \| P-Value \| \| --- \| --- \| --- \| --- \| --- \| --- \| \| GO_BP \| protein amino acid phosphorylation \| \| 136 \| 3.1 \| 2.60E-05 \| \| GO_BP \| intracellular signaling cascade \| \| 134 \| 3 \| 5.90E-05 \| \| GO_BP \| phosphorus metabolic process \| \| 178 \| 4 \| 6.40E-05 \| \| GO_BP \| phosphate metabolic process \| \| 178 \| 4 \| 6.40E-05 \| \| GO_BP \| mRNA processing \| \| 28 \| 0.6 \| 1.70E-04 \| \| GO_BP \| mRNA metabolic process \| \| 32 \| 0.7 \| 2.40E-04 \| \| GO_BP \| phosphorylation \| \| 149 \| 3.4 \| 3.90E-04 \| \| GO_BP \| transmembrane receptor protein tyrosine kinase signaling pathway \| \| 40 \| 0.9 \| 4.40E-04 \| \| GO_BP \| cell motion \| \| 55 \| 1.3 \| 5.20E-04 \| \| GO_BP \| neuron differentiation \| \| 53 \| 1.2 \| 5.30E-04 \| \| GO_CC \| ribonucleoprotein complex \| \| 73 \| 1.7 \| 2.10E-05 \| \| GO_CC \| cell leading edge \| \| 21 \| 0.5 \| 1.90E-04 \| \| GO_CC \| cell projection \| \| 53 \| 1.2 \| 5.60E-04 \| \| GO_CC \| chromatin remodeling complex \| \| 13 \| 0.3 \| 1.20E-03 \| \| GO_CC \| ribosome \| \| 43 \| 1 \| 3.10E-03 \| \| GO_CC \| nuclear lumen \| \| 85 \| 1.9 \| 4.20E-03 \| \| GO_CC \| organelle lumen \| \| 100 \| 2.3 \| 7.40E-03 \| \| GO_CC \| intracellular organelle lumen \| \| 100 \| 2.3 \| 7.40E-03 \| \| GO_CC \| membrane-enclosed lumen \| \| 102 \| 2.3 \| 9.60E-03 \| \| GO_CC \| intrinsic to endoplasmic reticulum membrane \| \| 11 \| 0.3 \| 1.10E-02 \| \| GO_MF \| protein kinase activity \| \| 132 \| 3 \| 1.40E-05 \| \| GO_MF \| enzyme binding \| \| 45 \| 1 \| 5.80E-05 \| \| GO_MF \| GTPase regulator activity \| \| 66 \| 1.5 \| 7.80E-05 \| \| GO_MF \| protein serine/threonine kinase activity \| \| 86 \| 2 \| 1.40E-04 \| \| GO_MF \| nucleoside-triphosphatase regulator activity \| \| 66 \| 1.5 \| 2.20E-04 \| \| GO_MF \| ATP binding \| \| 264 \| 6 \| 3.30E-04 \| \| GO_MF \| adenyl ribonucleotide binding \| \| 265 \| 6 \| 3.70E-04 \| \| GO_MF \| olfactory receptor activity \| \| 64 \| 1.5 \| 4.20E-04 \| \| GO_MF \| nucleotide binding \| \| 390 \| 8.9 \| 5.30E-04 \| \| GO_MF \| ribonucleotide binding \| \| 318 \| 7.2 \| 1.20E-03 \| |
| --- | --- | --- | --- | --- | --- | --- | --- | --- | --- | --- | --- | --- | --- | --- | --- | --- | --- | --- | --- | --- | --- | --- | --- | --- | --- | --- | --- | --- | --- | --- | --- | --- | --- | --- | --- | --- | --- | --- | --- | --- | --- | --- | --- | --- | --- | --- | --- | --- | --- | --- | --- | --- | --- | --- | --- | --- | --- | --- | --- | --- | --- | --- | --- | --- | --- | --- | --- | --- | --- | --- | --- | --- | --- | --- | --- | --- | --- | --- | --- | --- | --- | --- | --- | --- | --- | --- | --- | --- | --- | --- | --- | --- | --- | --- | --- | --- | --- | --- | --- | --- | --- | --- | --- | --- | --- | --- | --- | --- | --- | --- | --- | --- | --- | --- | --- | --- | --- | --- | --- | --- | --- | --- | --- | --- | --- | --- | --- | --- | --- | --- | --- | --- | --- | --- | --- | --- | --- | --- | --- | --- | --- | --- | --- | --- | --- | --- | --- | --- | --- | --- | --- | --- | --- | --- | --- | --- | --- | --- | --- | --- | --- | --- | --- | --- | --- | --- | --- | --- | --- | --- | --- | --- | --- | --- | --- | --- | --- | --- | --- | --- | --- | --- | --- | --- | --- | --- |

**Table S2 Distribution of intergenic DHSs overlapped with long noncoding RNAs (lincRNAs) in chicken MSB1 cell line.**

| Chromosomes | Peak_start | Peak_end | Peak_length | lincRNA_start^*^ | lincRNA_end^#^ | lincRNA_length |  | strand |
| --- | --- | --- | --- | --- | --- | --- | --- | --- |
| chr8 | 1946200 | 1946999 | 799 | 1937888 | 1969475 | 270 |  | + |
| chr8 | 1947200 | 1947999 | 799 | 1937888 | 1969475 | 270 |  | + |
| chr8 | 1947200 | 1948199 | 999 | 1937888 | 1969475 | 270 |  | + |
| chr8 | 1961200 | 1962199 | 999 | 1937888 | 1969475 | 270 |  | + |
| chr8 | 1966800 | 1967399 | 599 | 1937888 | 1969475 | 270 |  | + |
| chr23 | 1666400 | 1667599 | 1199 | 1654215 | 1684945 | 5473 |  | - |
| chr26 | 183600 | 183999 | 399 | 183621 | 184082 | 290 |  | - |
| chr27 | 3469400 | 3470199 | 799 | 3469551 | 3472836 | 1020 |  | - |
| chr28 | 4332000 | 4333399 | 1399 | 4306780 | 4376554 | 4488 |  | - |
| chr6_random | 31400 | 32799 | 1399 | 31347 | 33528 | 1711 |  | - |
| chr6_random | 31400 | 32999 | 1599 | 31347 | 33528 | 1711 |  | - |
| chrUn_random | 15083600 | 15085199 | 1599 | 15083545 | 15084849 | 1096 |  | + |
| chrUn_random | 15084600 | 15085599 | 999 | 15083545 | 15084849 | 1096 |  | + |
| chrUn_random | 23586200 | 23588599 | 2399 | 23585605 | 23586322 | 280 |  | - |
| chrUn_random | 48247800 | 48249399 | 1599 | 48218043 | 48291299 | 309 |  | + |
| chrUn_random | 48274000 | 48275599 | 1599 | 48218043 | 48291299 | 309 |  | + |
| chrUn_random | 48276600 | 48277599 | 999 | 48218043 | 48291299 | 309 |  | + |

* lincRNA_start: The start position of the first exon of this lincRNA in chicken galGal3 reference genome.

# lincRNA_end: The end position of the last exon of this lincRNA in chicken galGal3 reference genome.

**Table S3. The primer pairs for the validation of DHSs peaks and genes expression in MSB1 cells**

| Primer Name | Primer Sequence (5' to 3') | Primer Length (bp) | Validated Regions | Chrom | TSS | TES |
| --- | --- | --- | --- | --- | --- | --- |
| P1_F | CTGTGGTTTCGCTGGAGAGT | 20 | Peak1 | chr1 | 1.04E+08 | 1.04E+08 |
| P1_R | GACGCGATGTGATTTCTGCC | 20 |  |  |  |  |
| P2_F | CCCGGACATCTAAGGGCATC | 20 | Peak2 | chr1 | 1.04E+08 | 1.04E+08 |
| P2_R | GGCCGTTCTTAGTTGGTGGA | 20 |  |  |  |  |
| P3_F | CCTGCTCCCAAGACTGAAGG | 20 | Peak3 | chr2 | 44382000 | 44383399 |
| P3_R | AAGAGGACCTGGGAGAAGCT | 20 |  |  |  |  |
| P5_F | TCGTTTCTCTTTGGCCGGAA | 20 | Peak5 | chr3 | 84253400 | 84253999 |
| P5_R | ATGGTCCTCTTGTCGATGCC | 20 |  |  |  |  |
| NegPeak_F | TGTAAAACACTGCCCTCTGGT | 21 | Negative Control | chr11 | 11627400 | 11627999 |
| NegPeak_R | AAGAATGCAGGAGGGTGGC | 19 |  |  |  |  |
| G2_F | ATTGGAGGGCAAGTCTGGTG | 20 | ENSGALG00000021740 | chr1 | 1.04E+08 | 1.04E+08 |
| G2_R | CTCCCTTTCGATCGGCTGAG | 20 |  |  |  |  |
| G3_F | GCTCCTGAATTCACTGCTCCT | 21 | ENSGALG00000011951 | chr2 | 44378630 | 44383329 |
| G3_R | TAGTCCCAACCCACTCCGTT | 20 |  |  |  |  |
| G4_F | TTGGCCGGAAGAAAGAAGCT | 20 | ENSGALG00000001830 | chr28 | 1051659 | 1059655 |
| G4_R | AGGAACCTTTGCCCATCTCG | 20 |  |  |  |  |
| G5_F | AAGAACGGACACAAGGAGGG | 20 | ENSGALG00000015917 | chr3 | 84252819 | 84257463 |
| G5_R | TCTCGGATGGAACCTTTGGC | 20 |  |  |  |  |
| G10_F | CCACGTCTTTGAGGAGTCCC | 20 | ENSGALG00000023047 | chrZ | 71710287 | 71716732 |
| G10_R | GATCTGCCAGTGGTCGAACA | 20 |  |  |  |  |

TSS: Transcriptional Start Site;

TES: Transcriptional End Site.
