# Supplementary material for: Genome-wide mapping of DNase I hypersensitive sites and association analysis with gene expression in MSB1 cells
Source: Front Genet. 2014 Oct 13;5:308. doi: 10.3389/fgene.2014.00308 (PMC4195362; doi:10.3389/fgene.2014.00308)
Supplement: Supplementary file 2 [file DataSheet2.DOCX]

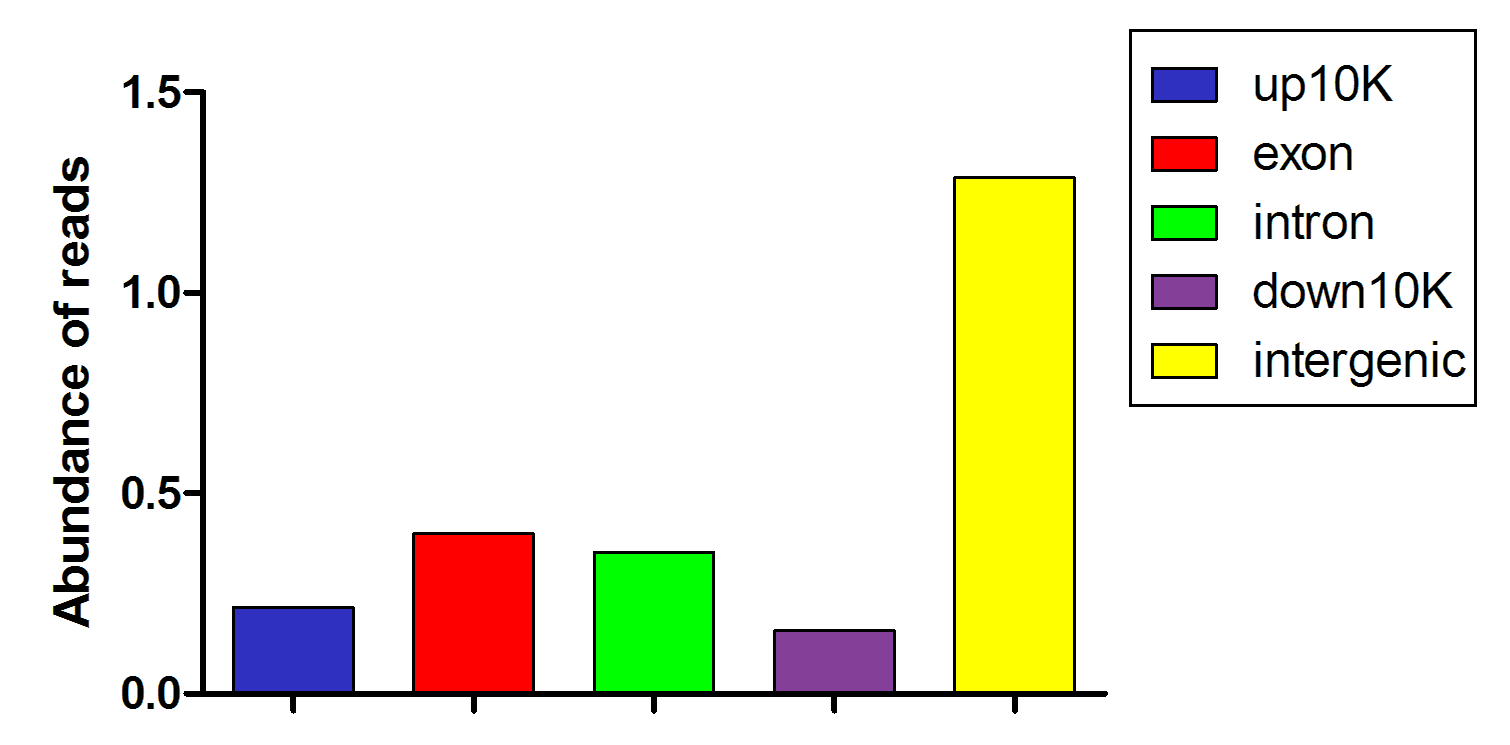


Figure S1. Sequence reads distribution of DNase I hypersensitive sites in MSB1 cell line. The percentages of reads distribution were normalized to the abundance values among different genomic regions.


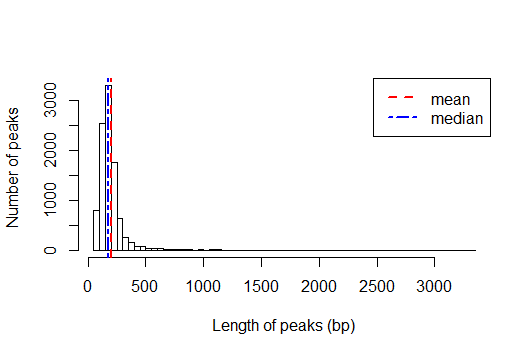


Figure S2. Length distribution of DNase-seq peaks identified by MACS 1.4.2. X-axis represents the length of peak; Y-axis represents the number of peaks.
